# Validation of a multiplex reverse transcription and pre-amplification method using TaqMan^®^ MicroRNA assays

**DOI:** 10.3389/fgene.2014.00413

**Published:** 2014-11-26

**Authors:** Joane Le Carré, Séverine Lamon, Bertrand Léger

**Affiliations:** ^1^Institute for Research in Rehabilitation, SuvaCare Rehabilitation ClinicSion, Switzerland; ^2^Centre for Physical Activity and Nutrition, School of Exercise and Nutrition Sciences, Deakin UniversityBurwood, VIC, Australia

**Keywords:** miRNA, qRT-PCR, multiplexing, plasma, human skeletal muscle

## Abstract

Since the discovery of microRNAs (miRNAs), different approaches have been developed to label, amplify and quantify miRNAs. The TaqMan^®^ technology, provided by Applied Biosystems (ABIs), uses a stem-loop reverse transcription primer system to reverse transcribe the RNA and amplify the cDNA. This method is widely used to identify global differences between the expression of 100s of miRNAs across comparative samples. This technique also allows the quantification of the expression of targeted miRNAs to validate observations determined by whole-genome screening or to analyze few specific miRNAs on a large number of samples. Here, we describe the validation of a method published by ABIs on their web site allowing to reverse transcribe and pre-amplify multiple miRNAs and snoRNAs simultaneously. The validation of this protocol was performed on human muscle and plasma samples. Fast and cost efficient, this method achieves an easy and convenient way to screen a relatively large number of miRNAs in parallel.

## BACKGROUND

MicroRNAs (miRNAs) are recently discovered small non-coding RNAs (∼22 nucleotides) regulating protein expression in animals and plants (Bartel et al., 2004). miRNAs can alter cellular function by binding the 3′-UTR of target mRNA and therefore inhibit the expression of the corresponding protein by either repressing protein translation or promoting mRNA degradation (Krol et al., 2010). miRNAs can be highly and specifically enriched in specific tissues and each miRNA can target multiple mRNA species (Lim et al., 2005; Sood et al., 2006). It is now well established that miRNAs play a pivotal regulatory role in many cellular processes including cell growth, proliferation, differentiation, and apoptosis (Bueno et al., 2008; Subramanian and Steer, 2010). miRNAs dysregulation is reflective of physiological and pathological adaptation processes and aberrant miRNAs expression is a hallmark of numerous disease conditions such as cancer, cardiovascular, neurological, and autoimmune disorders (Lawrie et al., 2008; Gidron et al., 2010; Caporali and Emanueli, 2011; Dai and Ahmed, 2011; Rasheed et al., 2013). Environmental factors including nutrition, sleep, exercise, hypoxia, and stress also contribute to the modulation of miRNAs expression (Chan et al., 2009; Wang and Cui, 2012; Zacharewicz et al., 2013).

Skeletal muscle is one of the largest organ of the body, making up approximately 40% of the whole body mass. Skeletal muscle is a highly plastic tissue able to adapt its size, structure and function in response to various internal and external stimuli, such as acute exercise, hypoxia, and training. miRNAs have been recently identified as novel, essential regulators of skeletal muscle health (Zacharewicz et al., 2013) and may account for specific regulation of muscle growth and differentiation (Buckingham and Rigby, 2014). miRNAs localization is, however, not restricted to cells and some miRNAs produced in cells are secreted in the bloodstream (Chen et al., 2008). miRNAs are reported to be highly stable in both plasma and serum (Mitchell et al., 2008) and circulating miRNAs expression is altered in pathological conditions. Although their role in circulation is not yet clear, miRNAs are promising biomarkers for the diagnostic of various pathologies, injuries and health conditions (Chen et al., 2008; Baggish et al., 2011; Zampetaki et al., 2012b).

The expression of specific miRNAs in tissues, including skeletal muscle and plasma, can be assessed using the reverse transcription quantitative real-time polymerase chain reaction (RT-qPCR). Mei et al. (2012) already described a broad range of commercially available miRNAs RT-qPCR assays. The classical approach involves the use of predesigned individual assays. The TaqMan^®^ technology provided by Applied Biosystems (ABI) uses a target-specific stem-loop RT primer that extends the 3′ end of the targeted miRNA to produce a cDNA template, which can then be amplified and quantified by real-time qPCR (Chen et al., 2005). This method is suitable for targeted quantification and validation of miRNAs profiling results. In contrast, miRNAs arrays collectively allow for the accurate quantification of 100s of miRNAs; a highly efficient method to establish the extended miRNAs expression profile of multiple tissue samples. However, miRNAs arrays are not suitable for the analysis of a small number of miRNAs on a large number of samples. Although the use of individual assays seems to be the most appropriate approach for this type of analyzes, the reverse transcription (RT) and quantification of each individual miRNA is time and reagent consuming. Recently, ABI described on their web site (Protocol for Creating Custom RT and Preamplification Pools using TaqMan^®^ MicroRNA Assays User Bulletin (Pub. no. 4465407 Rev. C) – cms_094060.pdf, 2014) a method allowing to multiplex the RT and pre-amplification (PA) steps. To our knowledge, this method was never validated in the literature. The aim of the present study was to validate and adapt this protocol on human muscle and plasma samples.

## DESCRIPTION OF METHODS

### METHOD DESIGN

**FIGURE 1:**
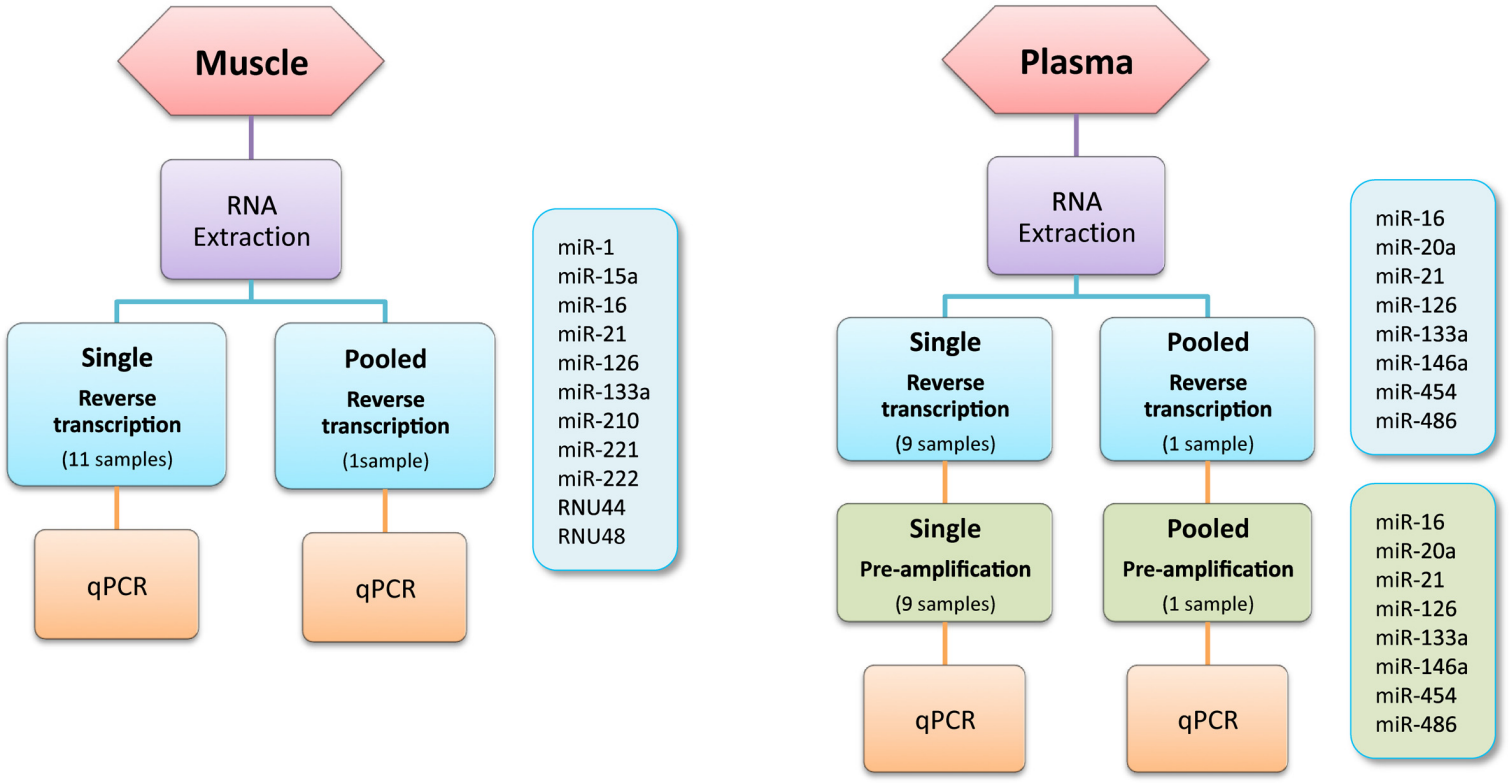
**Method design**.

In this report, we describe and validate a modified TaqMan^®^ Small RNA Assay method allowing the simultaneous RT followed by the PA of multiple miRNAs in both human muscle and plasma samples. The different steps of the method for each type of sample are depicted in **Figure 1**. The low amount of RNA in plasma justifies the addition of a PA step prior to the real-time qPCR to enhance the sensitivity of the reaction. Irrespectively of the tissue used ABI suggests to perform a PA step for any starting RNA amount smaller than 350 ng. Previous experiments from our lab demonstrated that a PA step is not necessary with skeletal muscle tissue as muscle miRNAs concentration is generally high enough to yield reliable results. We consequently decided to apply the PA step to plasma tissue only. The RT primer pool designed to analyze muscle and plasma miRNAs consisted in 11 (miR-1, miR-15a, miR-16, miR-21, miR-126, miR-133a, miR-210, miR-221, miR-222, RNU44, and RNU48) and 8 (miR-16, miR-20a, miR-21, miR-126, miR-133a, miR-146a, miR-210, and miR-454) miRNA specific primers sets, respectively. These miRNAs were selected on the basis of their relevance and expression levels in human muscle and plasma, respectively (Mitchell et al., 2008; Baggish et al., 2011; Davidsen et al., 2011; Zampetaki et al., 2012a; Aoi et al., 2013; Bye et al., 2013; Nielsen et al., 2014). In each type of sample, four miRNAs were further selected to complete the validation process.

### SAMPLES COLLECTION AND HANDLING

#### Muscle

Biopsies from the *vastus lateralis* muscle were obtained from four healthy subjects participating in a study previously published by our group (Faiss et al., 2013).

#### Plasma

Blood samples were obtained from four healthy subjects participating in a study previously published by our group (Faiss et al., 2013). Peripheral blood samples were collected from the antecubital vein in 2 × 2.6 ml EDTA tubes (Sarstedt *S*-Monovette) using a butterfly device. The samples were immediately centrifuged at 3500 *g* at 4°C for 10 min and the upper phase was collected. Plasma samples were then frozen in liquid nitrogen and stored at -80°C until further processing.

### RNA EXTRACTION

#### Muscle

Total RNA from skeletal muscle sample (approximately 25 mg of muscle) was isolated using a commercially available preparation, TriReagent^®^ (Molecular Research Center, Inc., Cincinnati, OH, USA), following the manufacturer’s instructions and as previously published by our group (Léger et al., 2006). Briefly, tissue samples were homogenized in 500 μl TriReagent^®^ with a power homogenizer (Polytron^®^ System PT2100, Kinematica AG, Lucerne) and incubated for 5 min at room temperature (R.T). Following this, 100 μl of chloroform were added and the sample was mixed during 20 s before being incubated for 10 min at R.T. After a 20 min centrifugation at R.T (12’000 *g*), 250 μl isopropanol were added to the aqueous phase and the sample was mixed for 20 s. Samples were precipitated overnight at -20°C and then centrifuged for 30 min at 4°C (12’000 *g*). RNA pellet was washed with 75% ethanol before being resuspended in 20 μl dH_2_O and stored at -80°C until use.

#### Plasma

Plasma aliquots were thawed on ice and centrifuged at 10’000 *g* for 10 min at 4°C to remove any remaining cellular contents. 400 μl of plasma were used for total RNA extraction using the mirVana PARIS kit (Life Technologies, Ambion, #AM1556) following the manufacturer’s protocol with minor modifications. Briefly, 400 μl of 2×denaturing solution and 800 μl of acid-phenol:chloroform were added to the plasma. Following this, the samples were vortexed for 60 s and incubated for 10 min on ice. After 20 min of centrifugation at 13’000 *g* at 4°C, the supernatant was collected and 1 ml of 100% ethanol was added. The lysate/ethanol mixture was then transferred onto a filter cartridge and centrifuged for 15 s at 10’000 *g* at R.T. Filter was washed and total RNA was eluted with 80 μl of pre-heated (95°C) elution solution provided by the manufacturer and then frozen at -80°C.

### REVERSE TRANSCRIPTION

#### Individual miRNA RT

miRNAs were reverse-transcribed using the TaqMan^®^ microRNA RT kit (Applied Biosystems, USA, #4366596) and the associated miRNA-specific stem-loop primers (TaqMan^®^ microRNA assay kit, #4427975). Total RNA from muscle was diluted at a concentration of 12.5 ng/μl and 4 μl of RNA were added to the reaction mix containing 0.15 μl 100 mM dNTP, 1 μl enzyme (50 U/μl), 1.5 μl 10× RT buffer, 0.19 μl RNase inhibitor (20 U/μl), 1.5 μl 5× RT specific-primer and 7.66 μl DEPC-treated water to obtain a final volume of 15 μl. The used primer concentration is twofold lower than the concentration recommended by ABI. In order to save reagent, we evaluated the possibility to reduce the primer concentration and tested the effect of a twofold primer dilution. Results showed that reducing the final concentration of primers only had a minor impact on the detection threshold, which increased from less than 0.5 cycles (data not shown). Thus, primer was used at a final concentration of 0.5× for all further analyzes. Concerning the RT of plasma miRNAs, due to the low plasma miRNA expression levels, a fixed volume of 3 μl of the 80 μl plasma RNA eluate was used as input in the reaction mix described above. RT reaction conditions were as follows: 30 min at 16°C to anneal primers, 30 min at 42°C for the extension of primers on miRNA and the synthesis of the first cDNA strand, 5 min at 85°C to stop the reaction. cDNA was then stored at -20°C until use.

#### Multiple miRNA reverse transcription

miRNAs were reverse-transcribed using the TaqMan^®^ microRNA RT kit (#4366596) and the associated miRNA-specific stem-loop primers (TaqMan^®^ microRNA assay kit, #4427975) with some modifications. A customized RT primer pool was prepared by pooling all miRNA-specific stem-loop primers of interest. In brief, miRNA-specific primers were pooled and diluted in 1× Tris-EDTA (TE) buffer to obtain a final dilution of 0.05× each. 6 μl of this mixture were added to the reaction mix containing 0.3 μl 100 mM dNTP, 3 μl enzyme (50 U/μl), 1.5 μl 10× RT buffer, 0.19 μl RNase inhibitor (20 U/μl) and 50 ng of muscle total RNA or 3 μl of plasma RNA. A final volume of 15 μl was reverse-transcribed with the following conditions: 30 min at 16°C to anneal primers, 30 min at 42°C for the extension phase, 5 min at 85°C to stop the reaction. cDNA was then stored at -20°C.

### PRE-AMPLIFICATION

In order to increase the amount of cDNA and to improve the sensitivity of the TaqMan^®^ qPCR reaction, a PA step was performed on plasma cDNAs. PA PCR conditions consisted in 10 min at 95°C, 2 min at 55°C, 2 min at 72°C, followed by 13 cycles of 15 s at 95°C, 4 min at 60°C and 10 min at 99.9°C. At the end of the run, the PA products were diluted 4× in 0.1× TE buffer pH 8.0 and stored at -20°C.

#### Individual cDNA pre-amplification mix

Protocol for the individual cDNA PA mix was as described by manufacturer. Briefly, the PA reaction combined 2 μl of RT product with 10 μl 2× TaqMan^®^ PreAmp Master Mix (#4391128), 2 μl 10× Megaplex Preamp primers V2.1 (#4399233) and 6 μl DEPC-treated water.

#### Multiple cDNAs pre-amplification mix

To simultaneously pre-amplify multiple cDNAs, we created a PA primer pool targeting the same miRNAs that were reverse-transcribed and containing 5 μl of each individual 20× TaqMan^®^ Small RNA Assay (part of #4427975) diluted in 500 μl 1× TE. The reaction mix was prepared by combining 3.75 μl of PA primer pool with 2.5 μl of RT product, 12.5 μl of 1× TaqMan^®^ Universal PCR MasterMix (2×), no UNG (#4440040) and 6.25 μl DEPC-treated water.

### REAL-TIME QUANTITATIVE PCR

All RT-qPCRs were carried out in triplicate with ABI products and were performed on the MX3000p thermal cycler system from Stratagene with the following conditions: one denaturing step at 95°C for 10 min, followed by 40 cycles consisting of denaturing at 95°C for 15 s and annealing and elongation at 60°C for 60 s, followed by an inactivation step of 10 min at 99.9°C. qPCR target sequences are provided in **Table 1**.

**Table 1:** List of TaqMan^®^ miRNAs and snoRNAs used in this study.

Name	Assay ID	Target sequence
hsa-miR-1	002222	UGGAAUGUAAAGAAGUAUGUAU
hsa-miR-15a	000389	UAGCAGCACAUAAUGGUUUGUG
hsa-miR-16	000391	UAGCAGCACGUAAAUAUUGGCG
hsa-miR-20a	000580	UAAAGUGCUUAUAGUGCAGGUAG
hsa-miR-21	000397	UAGCUUAUCAGACUGAUGUUGA
hsa-miR-126	002228	UCGUACCGUGAGUAAUAAUGCG
hsa-miR-133a	002246	UUUGGUCCCCUUCAACCAGCUG
hsa-miR-146a	000468	UGAGAACUGAAUUCCAUGGGUU
hsa-miR-210	000512	CUGUGCGUGUGACAGCGGCUGA
hsa-miR-221	000524	AGCUACAUUGUCUGCUGGGUUUC
hsa-miR-222	002276	AGCUACAUCUGGCUACUGGGU
hsa-miR-454	002323	UAGUGCAAUAUUGCUUAUAGGGU
hsa-miR-486	001278	UCCUGUACUGAGCUGCCCCGAG
hsa-miR-494	002365	UGAAACAUACACGGGAAACCUC
RNU44	001094	CCUGGAUGAUGAUAGCAAAUGCUGACUGAA
		CAUGAAGGUCUUAAUUAGCUCUAACUGACU
RNU48	001006	GAUGACCCCAGGUAACUCUGAGUGUGUCG
		CUGAUGCCAUCACCGCAGCGCUCUGACC

#### RT-qPCR with individual cDNA

Real-time PCR reactions were modified for a smaller final volume of 15 μl per well, using the same reagent proportions as recommended by the manufacturer. In each well, 1 μl of muscle cDNA or 1.5 μl of diluted PA plasma cDNA was added to the reaction mix containing 0.75× TaqMan^®^ Small RNA Assay (20×; #4427975) and 0.75× TaqMan^®^ Universal PCR MasterMix (2×), no UNG (#4440040). Plates were mixed by hand and briefly centrifuged before being loaded onto the qPCR machine.

#### RT-qPCR with multiple cDNAs

Real-time PCR reactions with multiple cDNAs were performed in a 20 μl final volume. A reaction mix containing 0.2 μl of multiplexed muscle cDNAs or 0.2 μl of pre-amplified plasma cDNAs, 7.5 μl 2× TaqMan^®^ Universal PCR MasterMix, no UNG (#4440040) and 11.3 μl DEPC-water was loaded in each well and 1 μl of 1× TaqMan^®^ Small RNA Assay (20×; #4427975) was added. The used MasterMix concentration is 25% lower than recommended by ABI. Previous experiments from our lab demonstrated that such reduction of the MasterMix concentration did not influence detection thresholds. Plates were mixed by hand and briefly centrifuged before being loaded onto the qPCR machine.

## RESULTS

### PRE-AMPLIFICATION STEP

As expected, the PA step significantly reduced CT detection threshold in both individual and pooled experiments. **Table 2** shows the CT values for the two conditions aforementioned and the differences in CT for the four miRNAs selected. In our hands we observed an average of 8.2 and 8.0 cycles difference for individual and pooled cDNA, respectively.

**Table 2:** Improvement of sensitivity through pre-amplification (PA) step on individual and pooled cDNA.

	Individual cDNA			Pooled cDNAs		
	w/o PA (CT)	With PA (CT)	Differencies	w/o PA (CT)	With PA (CT)	Differencies
miR-16	25.83	17.72	8.1	28.73	20.64	8.1
miR-20a	30.77	21.82	8.9	30.96	23.67	7.3
miR-146a	31.40	23.21	8.2	34.57	26.35	8.2
miR-454	32.53	24.89	7.6	35.05	26.66	8.4

### SENSITIVITY AND SPECIFICITY

**FIGURE 2:**
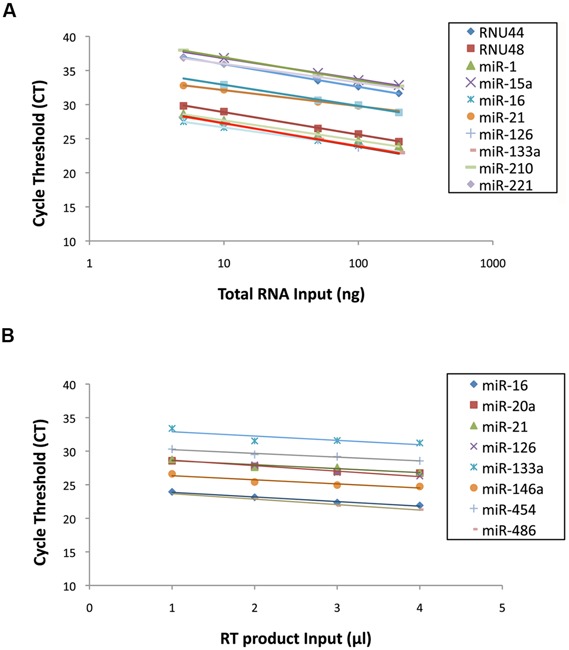
**(A)** Standard curves from 11 miRNAs expressed in human muscle tissue. **(B)** Standard curves from eight microRNAs (miRNAs) expressed in human plasma samples.

The sensitivity of miRNA quantification in muscle and plasma using the customized TaqMan^®^ miRNA Assay protocol was evaluated by a dose-response curve for each miRNA used in the RT primer pool. The RT primer pool for muscle analysis consisted in miR-1, miR-15a, miR-16, miR-21, miR-126, miR-133a, miR-210, miR-221, miR-222, RNU44, and RNU48. In the same manner, the RT primer pool for plasma analysis consisted in miR-16, miR-20a, miR-21, miR-126, miR-133a, miR-146a, miR-210, and miR-454. The protocol described in this manuscript is based on a fixed volume of RT product to be used in the PCR reaction. Therefore, increasing the cDNA input requires to increase the RNA amount used in the RT reaction. This explains why the standard curve was based on RNA dilution and not cDNA. A standard curve including 5 RNA dilution points (5, 10, 50, 100, and 200 ng of total RNA) was established for the miRNAs of interest present in muscle tissue. This represents a dilution of cDNA ranging from 0.067 to 2.6 ng. The low amount of RNA in plasma prevents standard RNA quantification via optical density measurement. Thus, a fixed volume of plasma RNA was used to perform a standard curve ranging from 1 to 4 μl of plasma RNA. All dilution points were reverse-transcribed separately before being amplified for each miRNA. All tested miRNAs were successfully amplified in both muscle and plasma. The efficiency and the coefficient of determination (R^2^) were determined from the standard curve for each miRNA present in the RT primer pool. The R^2^ of qPCR reactions on muscle samples were higher than 0.993 (**Figure 2A**) with efficiency ranged from 94.9 to 166.6%. The R^2^ of qPCR reactions on plasma samples were higher than 0.960 (**Figure 2B**) with efficiency ranged from 88.5 to 119.1% (**Table 3**). These results demonstrate a good linearity of the different assays and the fact that the first point of the standard curve is as low as 5 ng RNA (corresponding to predicted cDNA inputs of 0.067 ng for the multiplex method) indicates a high sensitivity of miRNAs amplification. The efficiency of these standard curves does not only reflect the specificity of qPCR primers but also the efficiency of the RT to convert RNA into cDNA. Indeed, standard curves do not result from a serial dilution of a single RT product, but from a serial dilution of a unique RNA sample, thus five RT products. This explains why some miRNAs have an excellent linearity despite of a reduced or increased efficiency. To verify that the efficiency of miR-21 (166.6%) in muscle was not related to the multiplexing method, we completed a standard curve following an individual RT of miR-21. Results showed an efficiency largely superior to 100% as well (142%, *R*^2^ = 0.95), suggesting that the efficiency of miR-21 in the multiplexing protocol is not related to the method. Together, these results revealed that the presence of multiple miRNA-specific stem-loop primers in the same RT reaction mix did not alter or inhibit the RT reaction by non-specific interactions. Moreover, the presence of multiple cDNA species in the qPCR reaction mix did not influence the specificity of the amplification. Furthermore, a no template control (NTC) was run for each miRNA to rule out cross contaminations of reagents or surfaces. No amplification curves were observed for any NTC, while all 60 assays were successfully amplified suggesting the absence of non-specific interactions.

**Table 3:** *R*^**2**^ and efficiency of the standard curves obtained with the modified TaqMan^®^ Small RNA Assay.

Muscle				Plasma		
	*R*^2^	Efficiency			*R*^2^	Efficiency
mir-1	0.999	117.9		miR-16	0.986	100.0
miR-15a	0.993	110.1		miR-20a	0.988	107.0
miR-16	0.999	133.5		miR-21	0.960	110.3
miR-21	0.994	166.6		miR-126	0.993	94.4
miR-126	0.999	94.9		miR-133a	0.996	88.5
miR-133a	1.000	99.0		miR-146a	0.967	109.4
miR-210	0.996	101.0		miR-454	0.994	119.1
miR-221	0.993	133.2		miR-486	0.998	96.6
miR-222	0.996	115.7				
RNU44	0.999	100.6				
RNU48	0.998	101.3				

*R^*2*^ value is the coefficient of determination of the standard curves and the efficiency is the rate at which the polymerase chain reaction (PCR) amplicon is generated. The *R*^*2*^ and the efficiency were calculated by the MxPro qPCR Software.*

### REPEATABILITY

Intra-assay variability was tested to assess the repeatability of the assay. Three independent RTs were performed from the same muscle RNA sample. For each aliquot, 11 muscle miRNAs (miR-1, miR-15a, miR-16, miR-21, miR-126, miR-133a, miR-210, miR-221, miR-222, RNU44, and RNU48) were simultaneously reverse-transcribed and then four miRNAs (miR-1, miR-16, miR-21, and RNU44) were amplified individually by qPCR. Similarly, one plasma sample was divided in three aliquots of 400 μl each and RNA was extracted as described. For each plasma sample, eight miRNAs (miR-16, miR-20a, miR-21, miR-126, miR-133a, miR-146a, miR-210, and miR-454) were simultaneously reverse-transcribed and then pre-amplified. Four plasma miRNAs (miR-16, miR-20a, miR-146a, and miR-454) were then analyzed individually by qPCR. The coefficient of variation (CV%) was calculated to determine the intra-assay variability. The CVs ranged from 0.27 to 0.38% and from 0.21 to 0.45% for muscle and plasma samples, respectively, indicating a high reproducibility of the assay (**Table 4**).

**Table 4:** Intra-assay repeatability of the modified TaqMan^®^ Small RNA Assay.

	Aliquot 1	Aliquot 2	Aliquot 3		
	CT	CT	CT	Average CT	SD	CV %
**Muscle**
miR-1	25.93	25.81	25.93	25.89	0.07	0.27
miR-16	26.58	26.41	26.53	26.51	0.09	0.33
miR-21	34.94	35.09	35.18	35.07	0.12	0.34
RNU44	33.98	33.88	34.13	34.00	0.13	0.38
**Plasma**
miR-16	21.94	21.77	21.78	21.83	0.10	0.45
miR-20a	26.45	26.24	26.26	26.32	0.12	0.44
miR-146a	26.69	26.65	26.58	26.64	0.06	0.21
miR-454	28.79	28.64	28.75	28.73	0.08	0.28

### ACCURACY

To assess the accuracy of the RT-qPCR system described herein, we show that the inter-subject expression variability for one specific miRNA is similar in the individual and pooled method. Therefore, we extracted RNA from four muscle and four plasma samples. Eight miRNAs used for the repeatability experiment in muscle (miR-1, miR-16, miR-21, and RNU44) and plasma (miR-16, miR-20a, miR-146a, and miR-454) were reverse-transcribed in each sample, once individually and once pooled in the same RT. After the amplification of these cDNAs, we observed that the individual cycle threshold (CT) values differed depending on the RT approach chosen. The use of reagents at different volumes and concentrations in the RT reaction explains these variations. It is however expected that the ratio CT (pooled cDNAs)/CT (individual cDNA) remains stable. The coefficient of variation (CV%) of this ratio was calculated for each reaction. The inter-assay CVs ranged from 0.5 to 2.3% and from 1.2 to 3.0% for muscle and plasma samples, respectively (**Table 5**), indicating that the CT value of the miRNAs amplified with the pool method accurately reflects the expected levels of expression.

**Table 5:** Coefficient of variation (CV%) of the ratio CT (pooled cDNAs)/CT (individual cDNA).

	Ratio CT pooled/individual method						
	Sample 1	Sample 2	Sample 3	Sample 4	Average	SD	CV %
**Muscle**
miR-1	1.43	1.38	1.42	1.42	1.41	0.02	1.8
miR-16	1.20	1.19	1.18	1.16	1.18	0.02	1.3
miR-21	1.36	1.38	1.33	1.31	1.35	0.03	2.3
RNU44	1.29	1.29	1.29	1.28	1.29	0.01	0.5
**Plasma**
miR-16	1.24	1.19	1.28	1.22	1.23	0.04	3.0
miR-20a	1.27	1.25	1.29	1.26	1.26	0.02	1.6
miR-146a	1.15	1.19	1.20	1.19	1.18	0.02	1.7
miR-454	1.12	1.12	1.14	1.14	1.13	0.01	1.2

## CONCLUSION

The individual analysis of multiple miRNAs is time and reagent consuming. Here, we describe an approach allowing to simultaneously measure multiple miRNAs in muscle and plasma samples. To our knowledge this method was never described in the literature whereas ABI published on their web site a protocol for creating custom RT pools using TaqMan^®^ microRNA Assays. In this way, we validate and bring minor adaptations to a technique allowing to multiplex the RT and PA steps. Validation results show that this customized method is not only sensitive and highly specific but also repeatable and accurate. In addition, the adaptations brought to the original protocol allow to save expensive reagents, such as stem-loop primers, MasterMix and PA mix, as well as time by eliminating the PA step for every tissue but plasma.

Lao et al. (2006) previously described an elegant and efficient multiplexing method based on TaqMan^®^ probes that allows the simultaneous amplification and analysis of up to 190 miRNAs. However, this method is restricted to the use of a pre-designed RT primer pool and therefore does not allow the multiplex analysis of novel miRNAs that may not be included in this pool. In contrast, our protocol allows to create a RT primer pool unique to the miRNAs of interest. Moreover, whereas Lao et al. (2006) results must be treated as microarray results and therefore require further confirmation by single PCR, the method we describe here does not require this step. Our method is therefore of high relevance to simultaneously analyze a limited number of miRNAs, selected from a previous microarray screening or from the literature, on a large number of samples. Altogether, it will facilitate the identification and measure of specific miRNAs biomarkers and greatly contribute to the understanding of how miRNAs regulate biological processes.

## Conflict of Interest Statement

The authors declare that the research was conducted in the absence of any commercial or financial relationships that could be construed as a potential conflict of interest.
